# Misinformation and de-contextualization: international media reporting on Sweden and COVID-19

**DOI:** 10.1186/s12992-020-00588-x

**Published:** 2020-07-13

**Authors:** Rachel Elisabeth Irwin

**Affiliations:** Department of Arts and Cultural Sciences, Lund University, Box 192, 221 00 Lund, Sweden

**Keywords:** News, Social media, Sweden, COVID-19, Pandemic, Policy evaluation

## Abstract

In the first month of the 2020 COVID-19 pandemic, Sweden took the same strategy as most other countries, working to “flatten the curve,” by slowing transmission so that the healthcare system could cope with the disease. However, unlike most other countries, much of Sweden’s implementation focused on voluntary and stepwise action, rather than legislation and compulsory measures, leading to considerable attention in the international media.

Six main narratives emerged in the international media reporting on Sweden during the first month of the COVID-19 pandemic: (1) Life is normal in Sweden, (2) Sweden has a herd immunity strategy, (3) Sweden is not following expert advice, (4) Sweden is not following WHO recommendations (5) the Swedish approach is failing and (6) Swedes trust the government. While these narratives are partially grounded in reality, in some media outlets, the language and examples used to frame the story distorted the accuracy of the reporting.

This debate examines the ways in which international media both constructs and represents a pandemic, and the implications for how researchers engage with news and social media. Cross-country comparison and the sharing of best practice are reliant on accurate information. The Swedish example underlines the importance of fact checking and source critique and the need for precision when presenting data and statistics. It also highlights limitations of using culture as an explanation for behavior, and the pitfalls of evaluating policy during a pandemic.

## Background

During the early stages of the 2020 COVID-19 pandemic, many governments and public health experts were focused on finding best practices in controlling the spread of the disease. Fact checking, source critique and precision in how data and statistics are presented are necessary preconditions for the sharing of cross-border experiences. As researchers we are also part of the media: we may be interviewed, we write opinion pieces and we share news on social media. We are reliant upon accurate information and we also have a responsibility for how we engage with the media.

Using the example of international reporting on Sweden’s COVID-19 response, in this debate I examine the ways in which the international media both constructs and represents a pandemic. One issue was that much of the media reporting was not fully accurate, hindering an in-depth discussion of best practice. Media reporting is also often at a superficial level and does not allow space for the details of policies or a consideration of regional or local experiences.

While there was a desire for facts and evidence, the frantic search for best practice also led some to draw premature conclusions. All of this misleading media coverage led to wasted time: Swedish representatives were forced to put significant energy into combating misinformation, rather than dealing with domestic challenges. A second issue was over the use of ‘culture’ as an explanation for policy choices. Culture and context certainly play a role in any country’s response; yet, a focus on culture can lead other countries to dismiss the lessons learnt from cultures that are seemingly unlike their own. A focus on culture can also lead to the exotification or simplification of a country’s response.

This article examines the first month of the 2020 COVID-19 pandemic. Sweden took the same strategy as most other countries, working to “flatten the curve,” by slowing transmission so that that the healthcare system could cope with the disease. However, unlike most other countries, much of Sweden’s implementation focused on voluntary and stepwise action, rather than legislation and compulsory measures (Table 1). This led to considerable attention in the international media, with the response gaining the moniker “the Swedish experiment.” [1] The suggestion that Sweden’s laissez faire response was synonymous with inaction became problematic enough for the Foreign Minister to dispute this on CNN [2].

**Table 1:** Public health measures taken in the first month of the response

General advice from the Public Health Agency (Folkhälsomyndigheten) as of 16 April 2020 • Stay home even if you feel the least bit sick. • Wash your hands frequently with soap and water for at least 20 s. • Keep away from others both indoors and outdoors. • Keep away from others on the bus, train, subway, tram and other public transport. • Avoid, funerals, baptisms, parties or weddings. • Keep away from others at sports venues, swimming pools and gyms and avoid changing in public changing rooms. • Do not travel during rush hour if you can avoid it. • Only travel if necessary. • If you are 70 years of age or older, it is of the utmost importance that you limit your social contacts and avoid places where people gather. Key restrictions and policy measures 11 March: Public events with more than 500 people banned (took effect 12 March). 16 March: Advice to work from home if possible. 17 March: Recommendation that senior high schools, universities and higher education move to distance learning (took effect 18 March). 19 March: Schengen-wide travel entry ban goes into effect. 24 March: Crowding in restaurant, cafes, and bars not allowed; table service should be utilized to minimize crowding at the bar. 29 March: Public events with more than 50 people banned (announced 27 March). 1 April: Further detailed guidelines and binding recommendations on physical distancing for individuals and for the public and commercial sectors released: for example, shops have a responsibility to rearrange their floor space to prevent crowding; public transport operators need to reduce crowding.	

In the book Good Sweden, Bad Sweden, journalist Paul Rapacioli describes how Sweden and Swedish values have been used as a weapon in a post-truth world [3]. Relatively few people have firsthand knowledge or experience with the country, and what they know tends to be positive and associated with so-called progressive values such as innovation, democracy, environmental concern and feminism. Sweden also ranks highly on most league tables. This means that negative stories are tantalizing, as they provide an exciting juxtaposition to the “lazy myth … that Sweden is some kind of paradise rather than a many-layered country of ten million people.”

Moreover, changes in how news is produced, circulated and consumed have also given rise to misinformation, disinformation and even so-called ‘fake news.’ Rapacioli was writing in the context of the migrant debate which characterized much of the 2010s, arguing that the far right was seeking to undermine progressive values by using selective reporting to ‘prove’ that migration was destroying Sweden. In international media, Sweden is often discussed as a “peculiar country in the north where everything is either perfect paradise or a collapsing hellhole.” [4] While the context of COVID-19 is different, many of the same mechanisms are at work here. This polarized view of Sweden is exemplified by two articles: three days before The Guardian reported that “Swedish PM warned over ‘Russian roulette-style’ COVID-19 strategy”, it had published a story covering southern Sweden’s emerging wine industry [5, 6].

In the media reporting of the “Swedish experiment” six main narratives emerged which I discuss in the sections that follow (1) Life is normal in Sweden, (2) Sweden has a herd immunity strategy, (3) Sweden is not following expert advice, (4) Sweden is not following WHO recommendations (4) the Swedish approach is failing and (6) Swedes trust the government. While these narratives are partially grounded in reality, in some media outlets, the language and examples used to frame the story distorted the accuracy of the reporting.

## Main text

I carried out a rapid review of mainstream Swedish and international news, focusing on the month following the WHO’s decision to characterize COVID-19 as a pandemic (11 March 2020) (Table 2).

**Table 2:** Media and internet sources reviewed

Online news	Television/video sources	Twitter hashtags	Google Search
**Swedish**	**Swedish Television** (via SVTPlay.se, Sweden Television's on-demand service**)**	#Tegnell	“Sweden’s COVID-19 Strategy”
Dagens Nyheter	Aktuellt (Daily news program)	#Coronasverige	
Svenska Dagbladet	Agenda (Sunday news program)	#SwedenInDenial	
Sydsvenskan	Public Health Agency Press Briefings (daily)	#CoronaVirusSverige	
The Local (in English)	Other (includes the Prime Minister’s speeches, ad hoc pres conferences and COVID-19 related news specials)	The Twitter hashtags and Google searches were used to identify more online news, including from the following sources: Al-Jazzera AFP Bloomberg Business Insider CNN.com Deutsche Wlle The Economist Euronews Foreign Policy Fortune Irish Times The New York Times Politico Reuters The Telegraph Time.com Sky News Vox	
Aftonbladet			
Expressen			
**International**	**International**		
The Guardian	WHO Media Briefings on COVID-19 (Mondays, Wednesdays and Fridays)		
BBC news (news.bbc.co.uk)			

I started with the websites of three Swedish morning newspapers: Dagens Nyheter and Svenska Dagbladet are national papers, while Sydvenskan is a regional newspaper for Skåne. I also reviewed the websites of the two main national evening papers: Aftonbladet and Expressen, and that of The Local, which produces English-language news in Sweden.

I watched COVID-19 related news on Sweden’s public service television channels (Sveriges Television, SVT), using the online streaming service SVTPlay.se. I watched the WHO’s press briefings every other day and the Swedish Public Health Agency (Folkhälsomyndigheten, FHM)‘s daily press briefings, as well as press briefings with the Prime Minster and other government representatives. I took notes and used SVTPlay to go back and transcribe key passages.

The State Epidemiologist at FHM, Anders Tegnell, was one of the most visible faces of the official response and drew considerable ire online [7]. I used Twitter to follow discourse on the Swedish response and to identify which news articles were being shared. Hashtags I reviewed were: #Tegnell, #Coronasverige, #CoronaVirusSverige and #SwedenInDenial.

Through Swedish media and social media, I identified which international news articles were being discussed in Sweden and reviewed these. I also used Google Search to look for further articles, using the search string ‘Sweden’s COVID-19 Strategy.’ Additionally, I read The Guardian and BBC News online every day. Most of the international reporting I discuss here comes from English-language sources, but I also reviewed articles from Danish, German and Norwegian sources. I created an archive of 88 screenshots, primarily from Twitter, 248 news articles and transcriptions from press briefings, which I organized by date. Although many of the media outlets I reviewed print physical newspapers, I reviewed only the online versions.

My approach to studying the media was inspired by ethnography. As a process, ethnography is about immersing oneself in a community, whether online or in real life. This was not a properly ethnographic study because I did not interact with people; rather I took an ethnographer’s view while consuming online media. A good ethnographer approaches the field with an open mind, and uses inductive and iterative approaches, akin to grounded theory; that is one does not go into data collection with a hypothesis, rather the themes, concepts and narratives are identified through immersion in the setting [8–10]. In this case, I immersed myself in online news media and social media, primary Twitter. As part of the analysis I drew upon narrative approaches from the social sciences: I examined how media outlets created an understanding of the Swedish approach through the ways in which they organized and presented facts and interview data.

While much of the international reporting of Sweden was balanced and mostly accurate, misinformation and de-contextualized truths wove in and out of the coverage, giving rise to further misinterpretation on social media. In the sections that follow, I discuss six of the main narratives of media reporting about the Swedish response during the first month of the pandemic. This debate provides a snapshot; that is, as the pandemic progressed the dominance of certain narratives waxed and waned, and some of the content changed. However, the early reporting set the tone for later news stories, so it is worth exploring this first month in detail.

### Narrative 1: life is normal in Sweden

While Sweden’s fellow Scandinavians and nearly all other Europeans are spending most of their time holed up at home under orders from their governments, Swedes last weekend still enjoyed the springtime sun sitting in cafés and munching pickled herrings in restaurants. Swedish borders are open (to EEA nationals), as are cinemas, gyms, pubs and schools for those under 16 [11].

International reporting painted a picture of Swedes eating at outdoor cafes and strolling in the sunshine, or even skiing [12, 13]. Trains and busses were ‘still shuttling people all over the country’ and cinemas were open [14]. ‘Sweden also keep domestic flights going, ‘despite the risk of spreading the coronavirus.’ [15] On the 9th of April, Vox reported that:

For months, the Scandinavian nation allowed large gatherings to form, schools for younger children to remain open, restaurants to serve late-night guests, and resorts to welcome thrill-seeking skiers [16].

The Vox article was striking, claiming that ‘for months’ Sweden had been acting normally. Yet, it was published less than a month after the WHO had characterised COVID-19 as a pandemic so most other countries had also been acting normal “for months.” One the first English-language articles to break the Swedish story was the aforementioned “Swedish PM warned over “Russian roulette-style’ COVID-19” strategy [5]. An early published version of the article stated that the Prime Minister, in a speech the night before had “urged Swedes to lunch at a local restaurant.” The text was later changed: Stefan Lövfen, in fact, had specified a “takeaway” lunch during a speech broadcast on the 22nd of March.

The narrative of Sweden as normal was misleading. The bulk of international media reporting focused on Stockholm, and most online videos showed the area around Kungsträdgården and Drottninggatan – the very city center. There was some truth to this narrative: during the second full week of April, it had been widely reported in national media that Stockholmers were pushing the limits of good advice, by sitting in crowded outdoor seating areas; the city of Stockholm responded by carrying out extra checks on restaurants and cafes over the Easter weekend [17]. However, central Stockholm is not representative of Sweden.

The main cinema chain in Sweden, Filmstaden, closed on the 17th of March so few people were attending the cinema when these articles were written [18]. After much discussion, SkiStar, the country’s main ski operator, decided to close its facilities on the 6th of April; ski gondolas were already closed on the 24th of March [ 19]. Sweden’s decision to keep a select few flights open was due to their designation as “societally important” routes: Visby is the main city on Gotland, an island. Kiruna is above the polar circle and the other cities: Luleå, Umeå, Östersund, Skellefteå, Örnsköldsvik, Sundsvall are also in the north and relatively remote. The government decided that flights needed to run not for tourists, but to ensure essential supplies and workers could reach these cities [20].

The wider picture – Sweden as normal – was misleading, because life was not normal. CNBC reported on a report released by Swedbank that forecast the economy would contract by 4% and that unemployment would reach 10% by the summer [21]. Already during the course of March 2020, the numbers of business going into administration (*konkurs*) had increased, with the hospitality industry seeing an increase of 1400% [22]. Stockholmers drinking in the sun should not symbolize the various Swedish COVID-19 experiences.

### Narrative 2: Sweden has a herd immunity strategy

Now they talk about Sweden, but Sweden is suffering very greatly. You know that, right? Sweden did that, The herd. They call it the herd. Sweden is suffering very very badly, it’s a way of doing it. -Donald Trump [23]

During this month, several Swedish researchers who disagreed with the government’s approach stated, both in national and international media, that the governments was following a herd immunity strategy. The herd immunity theory also forms a large part of the “Russian Roulette” article, which served as a basis for much of the English-language reporting, and the concept came up in most international reporting [16]. On the 9th of April, Vox published an article with the title and subtitle:

Sweden’s government has tried a risky coronavirus strategy. It could backfire. Critics say the government seeks “herd immunity” from the coronavirus. That could lead to more deaths.

The author tweeted the article with the text: It sure looks like Sweden is trying to achieve #herd immunity.”

The government and FHM denied multiple times that they were following a herd immunity strategy, with the Foreign Minister Ann Linde discussing this on Swedish television and CNN [2]. The herd immunity rumor has a complicated history. Mathematician Marcus Carlsson forwarded this theory in a series of YouTube videos and Annika Linde, the former state epidemiologist also promoted the theory that FHM’s strategy was based on herd immunity; she did this in a Facebook post which was picked up in the media [5, 24, 25]. State epidemiologist Anders Tegnell also mentioned the term often on television which further led to public confusion [26]. At other times his quotes were taken out of context. In an article in Svenska Dagbladet, which was re-quoted in international media, Tegnell stated that the main strategy was to have a slow transmission of COVID-19 so that the healthcare system could manage, but that herd immunity was “not contradictory” with this. He also said that herd immunity was a “great concept.” [24]

Part of the herd immunity rumor had to do with how Swedish implementation was described in international media as being drastically different than other countries. Anders Tegnell argued that critics were emphasizing the differences, rather than similarities of the approach [27, 28]. That is, Sweden was also trying to flatten the curve but without draconian measures. The government took this line not in order to promote herd immunity but because they did not think it realistic or healthy “to keep people inside for 4 or 5 months.” [29] That is, the government and FHM considered the level of restrictions put in place in the first month of the pandemic to be sustainable over the long term. Similarly, schools remained open in part because of the negative impact of closure on mental and physical health, and also because it would create a childcare problem, not least for key workers [30].

There was no evidence that FHM had a “secret” herd immunity plan, and it was never the ‘deliberate strategy’ of the government to ‘let the virus spread its course and spread through the population [31].’It is more accurate to state that there was a hope that some level of herd immunity would be a side effect of the approach, but it was not the main strategy.

### Narrative 3: Sweden is not listening to experts or data

At the end of March, a group of over 2000 individuals signed an open letter to the government asking for more measures. Time described them as ‘doctors, scientists and academics,” the Guardian as “doctors, scientists, and professors,” Vox as “academics and experts” and The Economist as ‘scientists and professors.” [11, 14, 16, 32] Both the Economist and the Guardian pointed out that Prof Carl-Henrik Heldin, chairman of the Nobel Foundation was on the list. To support this statement Time and Vox linked to The Guardian’s live reporting from 24 March in which Jon Henley had written that “More than 1,500 medical and other academics in Sweden ranging from full professors to post-doc researchers have signed a petition calling on the government to change its coronavirus strategy.”

There are several problems with this letter. Although there are a number of well-regarded public health experts on the list, the six main authors, while professors, did not work in public health. This was something pointed out by Dagens Nyheter, while international media ignored this [33]. Moreover, there were many masters and PhD students on the list, as well as a handful researchers outside of Sweden who signed the letter. These are hopefully the experts of the future, but not necessarily current experts.

The letter was not widely circulated. The copy of the letter and list I saw was tweeted by Sten Linnarsson on the 26th of March and then retweeted by Nils-Göran Larsson, both of whom were signatories. To my knowledge, the only media source which has shown the letter was Vice in a video [34]. The first paragraph of the English version, as displayed by Vice was:

The Swedish government must act not (sic) to step up the fight against COVID-19

We have signed this open letter to request the Sweden Government immediatley (sic) take measures to follow the recommendations of the World Health Organization (WHO) to their full extent, including interventions to reduce the mobility and contact in the population and to support and incorporate the latest technologies to increase our capacity to test for Covid-10 (sic) infections nationwide.

The letter’s starting point was that the Swedish government had not followed WHO Recommendations (I discuss this narrative in the following section). The second paragraph of letter also perpetuated the rumor that Sweden was following a herd immunity strategy.

A second example comes from media reporting of a piece in the Lancet. According to AFP and Euronews, respectively:

“A study published last week in the medical journal *The Lancet*, titled “COVID-19: Learning from Experience,“ said that the “initial slow response in countries such as the UK, the USA, and Sweden now looks increasingly poorly judged.” [35]

“A study published in the medical journal, The Lancet, indicated that “the initial slowness of reaction from countries such as the UK, the US and Sweden now appears to be increasingly unwelcome” [36].

The ‘study’ was in fact an editorial written by the Lancet’s editor-in-chief Richard Horton [37]. Richard Horton is immensely influential in global health and no doubt could be described as an expert. However, this was his opinion, not a study, even though it was reported as such.

These examples aside, there were other instances of researchers, including those with backgrounds in virology, epidemiology and public health who raised valid concerns about how FHM has handled the evidence. An interesting question revolved around modelling done by Imperial College. This included the infamous work that, according to media reports, pushed the United Kingdom to change its COVID-19 strategy, although Anders Tegnell has argued that the British media have overstated the importance of the research in its policy decisions [38]. Within the debate pages of Swedish newspapers, researchers discussed the strengths and weaknesses, scope and applicability of the Imperial College modelling to the Swedish context [39–42]. FHM also publicly discussed their consideration of the evidence [43].

Some Swedish researchers also accused the Swedish government of only looking at the economy, with one writing in a leaked email chain “How many lives are they prepared to sacrifice so as not to ... risk greater impact on the economy? [5, 11] Another researcher was cited several times as stating that the government was not using scientific evidence in their decision making, stating: “We don’t have a choice, we have to close Stockholm right now.” [16, 32, 44, 45]

Modelling is not evidence, and models are only as good as the assumptions and data that is fed into them. Professor David McCoy discussed eloquently in The Guardian how the health and socioeconomic impacts of various lockdown measures had not been modelled [46]. I would suggest that the Swedish government and authorities did not take into account “the economy” but were cognizant that there are long and short term health effects of unemployment and underemployment. Additionally, the choice to ‘lockdown’ an entire community was also an experiment taken in the absence of definitive evidence, and many types of ‘lockdown’ measures, such as the quarantining of a whole city are not possible under Swedish law [47, 48]. Yet, international press focused on the conflict between FMH and other researchers, rather than fundamental questions about the limits and potential of mathematical modelling, and its role in policy-making. This was a missed opportunity to educate readers.

I return to the scope of evidence in the conclusion, but offer a further example here: On the 10th of March, FHM recommended against ‘unnecessary’ visits to care facilities (*äldreboende*) and the government implemented a ban on the 1st of April. Care facilities are run by local municipalities and in practice many municipalities had already banned visits before the 1st of April. In Norway, municipalities implemented different types of bans at different times, many later than in Swedish municipalities, with a national ban coming into force on the 7th of April. Yet, already by the 7th of April, FHM had reported that COVID-19 was present in half of the care homes in the city of Stockholm [49]. Undertaking serious analysis in the middle of a pandemic is challenging, but an initial comparison between care homes in Norway and Sweden suggest that simply banning visits is not an easy solution because it did not work in Sweden.

### Narrative 4: Sweden is not following WHO recommendations

The argument that Sweden is not following WHO recommendations came up in several debate articles in Swedish news [42, 50]. Internationally, Time reported that “experts say the Swedish government is not following the World Health Organization.” [32] CNN included a quote from a spokesperson from the WHO Regional Office for Europe who had said “it’s imperative” that Sweden “increase measures to control spread of the virus, prepare and increase capacity of the health system to cope, ensure physical distancing and communicate the why and how of all measures to the population.” They also included a link to a Tweet by WHO/Europe from 8 April which read “There is a fresh surge [of cases] in Sweden.” It also found in the letter written by over 2000 individuals, as noted in the previous section. There are two issues with this line of argument. Firstly, there is a general misunderstanding about what WHO recommendations are and are not, and secondly, I argue that Sweden followed the WHO’s ‘recommendations’ regarding public health measures [51].

The WHO issues many types of documents, such as recommendations, advice, or technical guidance. Articles 19–23 of the WHO’s constitution cover the legal basis for recommendations, conventions, agreements and regulations. What people think of as “WHO recommendations” are usually a mosaic of binding and mostly non-binding policy, advice, technical guidance and even international law and commercial law. A ‘recommendation’ found in an Interim Guidance document is completely different than a ‘recommendation’ endorsed or adopted by the World Health Assembly. Many commentators took Dr. Tedros’s mantra “test, test, test” to be a WHO recommendation that all countries should test everyone. A more accurate interpretation of the WHO’s Interim Guidance is that testing needs to be carried out with regard to a county’s capacity, context and situation [52].

In general terms, the WHO provides guidance to 194 Member States, which means that WHO ‘recommendations’ are rather vague. Most of the time, the WHO provides a menu of policy options from which countries can adapt or implement measures to take based on their national and local contexts. In Interim Guidance issued on the 7th of March, the WHO provided recommendations for public health measures, writing the countries should ‘consider, based on local and/or global evaluation’ the following: avoid crowing, school closures, public transportation and workplace closures and public health quarantine [53]. FHM did consider these, and adapted WHO recommendations in line with the Swedish context.

Another example comes from the International Health Regulations (IHR). The WHO declared COVID-19 a public health emergency of international concern on the 30th of January and issued temporary recommendations under the IHR. Some temporary recommendations under the IHR are binding, such as the obligation to share information with WHO, but most are not binding. In general, the WHO prefers that countries avoid border closures, in part because the disruption they cause can prevent medical supplies from reaching populations in need; also border closures are not usually that effective. Specifically, under Article 43 of the IHR:

States Parties implementing additional health measures that significantly interfere with international traffic are obliged to send to WHO the public health rationale and justification within 48 h of their implementation. WHO will review the justification and may request countries to reconsider their measures. WHO is required to share with other States Parties the information about measures and the justification received [54].

Sweden did not close its borders on its own but was forced into the Schengen-wide border closure.

WHO advice, technical guidance and recommendations are extremely important, not least in a pandemic situation and countries should take seriously documents issued by the WHO. It is important to understand that most ‘recommendations’ are not universal but are written in a way which allows countries to adapt them to their own contexts. Whether or not Sweden’s interpretation of WHO ‘recommendations’ in this instance was effective or appropriate is a different question; but Sweden followed WHO ‘recommendations’ with regard to public health measures.

This distinction matters: on the 21st of May 2020, the sustainability rating company Standard Ethics, downgraded Sweden’s rating having assessed that: “during the first phase of the COVID-19 pandemic, Swedish health policy did not comply with World Health Organization recommendations.”

### Narrative 5: the Swedish approach is not working; and Sweden has or will change course

On the 3rd of April, Reuters led with “Sweden’s liberal pandemic strategy questioned as Stockholm death toll mounts,” in which they wrote about a “spike in novel coronavirus infections and deaths.” [44] The Business Insider reported that “Sweden, which refused to implement a coronavirus lockdown, has so far avoided a mass outbreak. Now it’s bracing for a potential surge in deaths.” [55] On the 4th of April Bloomberg noted that the number of Swedish deaths rose to 373 on Saturday, up 12% from Friday [56]. On the 9th of April, Time Magazine reported that “Sweden’s relaxed approach to the Coronavirus could already be backfiring,” using the case fatality rate as part of the argument, although they recognized the challenges of this metric [32]. On the 4th of April, Deutsche Welle reported that “Sweden mulls U-turn on coronavirus restrictions.” [57]

Part of this reporting originated in a bill (*proposition*) proposed 7th of April (but which had been discussed in different forms during the previous days). This bill gave the government additional powers under the Communicable Diseases Act (*Smittskyddslagen*) to implement so-called ‘lockdown’ measures, such as closing harbors, train stations and shopping malls [58]. The interpretation in much of the international media was that Sweden was changing course. However, the Irish Times was one of the most accurate accounts:

“Sweden has reached cross-party agreement on sweeping emergency powers to close shops, restaurants and schools in response to the coronavirus crisis – but the government has no immediate plans to use them.” [59]

That is, during the course of the pandemic it became clear that existing legislation did not allow the government to act quickly in taking more ‘lockdown’-type restrictions, but there will be no immediate plans to use the measures [60]. In a press conference The Prime Minister referred to these new measures as ‘tools in a toolbox.’ The idea was not that Sweden was changing course but wanted to be prepared to face future uncertainties caused by COVID-19.

The effectiveness of the Swedish approach is difficult to fully assess at the time of writing, which I return to in the conclusion. Already after the first month of the pandemic, Sweden had seen a decrease of seasonal influenza and calicivirus disease (*vinterkräksjukan*) which indicated that voluntary social distance measures were working [61, 62] People’s behavior had also changed, although perhaps not as quickly as it could have [63]. Additionally, the pandemic curve in Stockholm was ahead of the rest of the country, making it difficult to draw conclusions on Sweden overall. It is also challenging to compare cross-country data, especially when journalists are not precise about what numbers are being reported: case fatality rates, total deaths, deaths per 100,000 or excess mortality. Other newspapers compared Sweden to the other Nordic countries, without noting that Sweden’s population is roughly twice as large as Norway, Denmark, or Finland. Although these countries later diverged in the number of deaths per 100,000, during the first month of reporting Denmark and Sweden had similar rates, so this omission distorted the early picture.

If more stringent measures are put in place, then they may be reported as ‘Sweden changing course’ or ‘the Swedish approach is failing.’ However, as early as the 22nd of March in an address to the nation, the Prime Minister was very clear that more measures should be expected; and the Public Health Agency stressed repeatedly that they were are taking a stepwise approach: “the right measure at the right time.” Similarly, when some travel restrictions for the summer were lifted in June, the government was clear that restrictions would be put back in place if the epidemiological situation changed [64]. If the government puts in more measures, it will be in line with their actions and the advice of FHM.

As a follow-up example, in June 2020 Anders Tegnell gave a short interview to Sveriges Radio in which he stated that there was ‘room for improvement’ in the Swedish approach and that, if the virus were encountered today, with the knowledge that we now have, that Sweden’s approach would have been between what the country did and what other countries did [65]. This was picked up in the international media with headlines such as “Sweden chose a looser lockdown. The scientist behind the strategy now says the death toll is too high” and “Architect of Sweden’s coronavirus strategy regrets not imposing tougher lockdown.” [66, 67] Tegnell clarified his statement the following day, as reported in The Local:

We still think that the strategy is good, but you can always make improvements, especially when looking back. I personally think it would be rather strange if anyone answered anything else to such a question. You can always do things better,” he said, adding that he did not necessarily think he had been misquoted, but that his comments had been overinterpreted [68].

### Narrative 6: Swedes trust the government

The final narrative returns us to some of the points raised in Good Sweden, Bad Sweden. Much of the early international coverage focussed on high-levels of trust in Sweden [32]. Historian Lars Trägårdh explained it accurately for several papers, noting that citizens trust public institutions and the government, the government trusts the people to do the right thing, and there is social trust amongst citizens as well [11, 12, 69].

As part of this is the cultural code of recommendations and advice. The government expects that Swedes understand that recommendations are to be followed, they are “not just loose tips,” as the Prime Minister clarified in a speech. Representatives from FHM repeatedly noted that public health measures, including infectious disease control, are based on “voluntary measures, acceptance and understanding in the population - that the Swedes trust and follow recommendations from their authorities.” [70]

By most quantitative and qualitative measures, there are high levels of trust in Sweden. However, in describing the Swedish approach, national myths blend with truths. In addition to the “Swedes trust authority” discourse there are also a number of ideas circulating about how Swedes are. Writing for Politico Lisa Bjurwald, herself Swedish, argues that Swedes naturally socially isolate, writing “Skype-based relationships? No hugging? For Swedes, that’s not social distancing. That’s just life.” [71] Carl Bildt, a former prime minister joked to the Economist that, “Swedes, especially of the older generation, have a genetic disposition to social distancing anyway [11]. On social media, people have questioned whether or not Swedes are responsible, citing the existence of the state-owned liquor store Systembolaget as evidence. This is the narrative that Swedes cannot control their alcohol intake and must have alcohol sales restricted. Even the term ‘peace-damaged’ (*fredsskadat*) was discussed in national media [72]. Simplified, this is the idea that, because Sweden has not been at war for over 200 years, it is difficult for people to imagine bad things happening on a large scale on Swedish soil, and that is why Sweden did not act as forcefully as other countries.

A related narrative, found more in social media than in the news, is that Swedes blindly trust authority, or that “trust seems to be equated with not questioning.” [73] Some of this has come from foreign residents, who are not used to the Sweden system or feel that Swedes are dismissive of their concerns [74]. Similarly, Swedes have been described as arrogant or even post-colonialist in their approach to non-Swedes living in Sweden [74, 75].

Yet, a country cannot be reduced to a few cultural stereotypes, and distilling an entire country into a set of traits and values also ignores complexity. It is not simply the Swedes have high levels of trust and ‘know’ how to follow recommendations. Much of the government and authority’s response also has to do with the legal system and history of Swedish administration, the latter of which gives a great deal of independence to agencies such as FHM [12, 47, 48, 76]. There are other contextual factors that impact the spread of disease, which have been noted by some articles. Sweden has a low population density compared to most other European countries and more than half of households are single-person (https://ec.europa.eu/eurostat/web/products-eurostat-news/-/DDN-20170905-1?inheritRedirect=true).

Focusing on culture also leads to constrained thinking, in which commentators find it difficult to explain ‘Stockholmers drinking beer in the sun’ or people ignoring recommendations, without concluding that assumptions of ‘Swedish responsibility’ are false. One can also become stuck in a cycle of criticizing Swedish ‘culture.’ In setting health policy, there is a fine line between taking context into account and reducing behavior to ‘culture,’ as if it is a fact or a variable to be plugged into a model. Rather, history, law, socioeconomic factors and tradition have created a tangled ‘web of significance’ – to borrow from anthropologist Clifford Geertz – in which different Swedes make decisions and live their lives.

## Conclusions

Not all reporting is misleading, and there were a number of balanced articles in the month following the WHO’s characterization of COVID-19 as a pandemic. Several of the ‘foreign’ reporters were Swedish or had lived in Sweden for years, spoke the language and could contextualize the reporting. It is not uncommon that an editor changes text and headlines, so the blame for sensationalizing does not always lay on the journalist. Also, owing in part to changes in the news industry over the past two decades, working conditions can be very difficult, especially for freelance journalists.

Moreover, a journalist or newspaper cannot always control how a story is interpreted and shared on social media or in the online comments sections. For instance, several newspapers covered a statement from the Italian ambassador in which he stated that the WHO ranked the Italian health system as the second best in the world, while Sweden’s was 23rd [77]. This statement became truth on Twitter. Very few noted that this ranking was from the 2000 World Health Report, itself a controversial report [78]. Additionally, the interplay between social media and news media amplifyies bias, which feeds back into reporting.

I also do not wish to criticize individual researchers – people’s complex arguments can be turned into soundbites when they are interviewed in the news; this is why it can be helpful to attend media training offered by many universities. However, while most articles are balanced, the negative ones are shared more widely [79]. This can have a negative impact upon public health action and can hinder cross-border comparisons.

Both the head of FMH, Johan Carlson and the State Epidemiologist, Anders Tegnell argued multiple times that the Swedish implementation of COVID-19 measures was not that different, but rather it was the ‘rhetoric and language’ that differed [80]. Interviewed in The Local, science journalist and epidemiologist Emma Frans noted that journalists often look for conflict, which is problematic in a pandemic:

“It’s a different issue compared to other kinds of news reporting. You have to be very responsible and it’s very important to get things right,” she says. “In a democracy you should always be able to question authorities, but with disease outbreaks, it’s also important that people trust the authorities and listen to their advice otherwise we get problems [81].

While I have focused on how international media has reported on Sweden’s handling of the COVID-19 pandemic, these findings raise broader questions on how the media reports on all country responses. Firstly, fact checking and source critique in a pandemic is important. Two characteristics of the international reporting was that many of the articles cited previous articles without checking the source or that they reported ‘expert’ opinion as scientific truth. The reporting was also not always discerning about defining concepts such as expertise, facts, data and science.

Secondly, some international media failed to communicate the complexities of science and policy, especially in the first month of the pandemic. For instance, terms like “surge” or “dramatic increase” are neither precise nor helpful. The line “the death toll keeps rising” is particularly useless because the number of deaths will rise until the disease is eliminated; it clarifies nothing about changes in the number of daily deaths. A ‘spike’ in cases can be a data artefact; it can be a result of delays in reporting or cases getting ‘stuck’ in the system before it can be updated. Other metrics, such as the number of people in intensive care, may also be useful. The idea of looking at excess mortality barely surfaced in mainstream media. Different countries are at different places in their pandemic curve, and it is difficult to draw immediate conclusions on cross-country data. Most science and health correspondents are well versed in interpreting findings and metrics. However, because there was such demand for COVID-19 context, some newspapers relied on journalists not used to interpreting public health policy interventions and graphical displays of data.

Thirdly, country-wide statistics and narratives are misleading. In the first month of the pandemic, the majority of cases and deaths were in greater Stockholm. The pandemic played out very differently in other parts of the country, both in terms of statistics but also behavior.

Fourthly, the use of ‘culture’ as an explanation also has its limits. When culture is used as an explanation, it can be easy to dismiss a country’s response as too contextual. While policies cannot be copied and pasted between countries, the lessons learnt from policy failures and successes are useful. Another challenge is that when culture is reduced to a soundbite, it can be tempting to focus on exotic practices or to judge people’s beliefs and traditions as negative. That is, culture can become a scapegoat for ‘harmful behavior.’ The reality is that culture is somewhat ephemeral, it exists alongside tradition, law and is shaped by socioeconomic forces.

Media is how we know what we know; it constructs reality. Journalists and editors choose what stories are told and what stories are not told [82]. Fake news is rarely totally fake and this matters for all of us. As Paul Rapacioli has noted: “When it comes to news, truth is context. A lack of context doesn’t make a story false but it greatly reduces the truth of it.” [3] Cross-border comparison is reliant on accurate information. All countries, particularly those in which the pandemic has not yet peaked, look for information on best practice. How we can learn from each other when we do not have the facts straight?

Finally, none of us are experts in all aspects of science and as researchers and lectures we depend on good reporting for understanding the world, and for communicating it to our students. As researchers we are also part of the news. We all have a responsibility to act on our conscious and speak out if we have a scientific basis for questioning authority. We have a responsibility to speak within the bounds of our expertise and to present facts, not suppositions. We have a responsibility to be critical of sources, both scientific and non-scientific. As many researchers are active on social media, we also have a responsibility to share accurate information and news.

### A final word on evaluating policy in a pandemic

It is already clear that there were problems with the Swedish public health response to COVID-19, not least that the government and FMH, like many other countries, underestimated the risk of COVID-19 spreading outside of Asia. Early reporting also identified class-based differences in mortality and morbidity patterns [83].

Taking the wider perspective, the success or failure of the so- called “Swedish experiment” may never be definitely proven. Researchers will be analyzing the data on COVID-19 for years and evaluating policy measures is challenging. While it may be relatively easy to compare the number of deaths directly attributable to COVID-19 there are many other metrics to consider in calculating the overall burden of disease. One also needs to look at the demographics of the spread, and to consider regional differences when analyzing epidemiological patterns.

There are also downsides, particularly in places with more stringent 'lockdowns.' Already in the first month of the pandemic, alarms were raised over the risks living with an abuser in ‘lockdown’ and of increased alcohol misuse. What will be the cost to health of the lost wages and unemployment affect health outcomes? What is the short and long-term impact of limited exercise and confinement, postponed health and dental care? Many of these challenges disproportionally affect already disadvantaged communities, and particularly low and middle-income countries. The response to COVID-19 may potentially exacerbate inequalities for generations to come.

Public health is not about the individual case. Arguably it is not even about the specific disease. Rather, public health is a about the whole picture. Here we can invert the question about the Swedish ‘experiment’: What is the real experiment, and what do we have evidence for or against: living in a state of semi-physical distancing or locking down entire countries? The answer is that we do not know yet.

## Data Availability

All the online news sources are available online, although some are behind paywalls. Most television news from SVT is available for a week on SVTPlay.se. Researcher can request access to the television archives upon request.
